# Dose–response curve slope helps predict therapeutic potency and breadth of HIV broadly neutralizing antibodies

**DOI:** 10.1038/ncomms9443

**Published:** 2015-09-29

**Authors:** Nicholas E. Webb, David C. Montefiori, Benhur Lee

**Affiliations:** 1 Department of Microbiology, Immunology and Molecular Genetics, University of California, Los Angeles, California 90024, USA; 2 Department of Surgery, Duke University Medical Center, Durham, North Carolina 27710, USA; 3 Department of Microbiology, Icahn School of Medicine at Mount Sinai, New York, New York 10029, USA

## Abstract

A new generation of HIV broadly neutralizing antibodies (bnAbs) with remarkable potency, breadth and epitope diversity has rejuvenated interest in immunotherapeutic strategies. Potencies defined by *in vitro* IC_50_ and IC_80_ values (50 and 80% inhibitory concentrations) figure prominently into the selection of clinical candidates; however, much higher therapeutic levels will be required to reduce multiple logs of virus and impede escape. Here we predict bnAb potency at therapeutic levels by analysing dose–response curve slopes, and show that slope is independent of IC_50_/IC_80_ and specifically relates to bnAb epitope class. With few exceptions, CD4-binding site and V3-glycan bnAbs exhibit slopes >1, indicative of higher expected therapeutic effectiveness, whereas V2-glycan, gp41 membrane-proximal external region (MPER) and gp120–gp41 bnAbs exhibit less favourable slopes <1. Our results indicate that slope is one major predictor of both potency and breadth for bnAbs at clinically relevant concentrations, and may better coordinate the relationship between bnAb epitope structure and therapeutic expectations.

## Abstract (web summary)

Potencies of HIV broadly neutralizing antibodies are usually defined by their *in vitro* IC_50_ and IC_80_ values, but much higher levels will be required for successful immunotherapies. Here, Webb *et al*. predict antibody potency at therapeutic levels by analyzing dose–response curve slopes, which correlate with epitope class.

## Graphical abstract

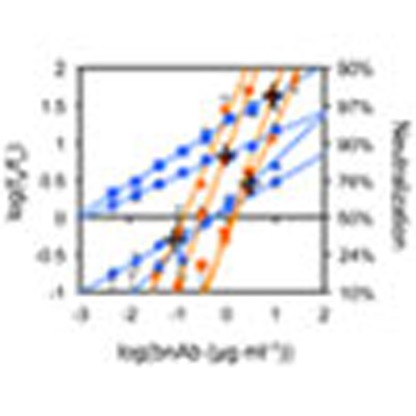

Several regions of the HIV-1 envelope glycoprotein spike are vulnerable to broadly neutralizing antibodies (bnAbs); these regions include the CD4-binding site (CD4bs) of gp120 (refs 1, 2, 3, 4), glycan-dependent epitopes in the second and third variable regions (V2 and V3) of gp120 (refs 5, 6, 7, 8), linear epitopes in the membrane-proximal external region (MPER) of gp41 (refs 9, 10, 11) and glycan-dependent epitopes that bridge gp120 and gp41 (refs 12, 13, 14, 15). This assortment creates opportunities for combinations of bnAbs to target multiple epitopes in an effort to achieve optimal coverage and impede escape^16^. Indeed, the identification and characterization of these bnAbs has generated renewed optimism that novel vaccines can be designed to elicit similar types of antibodies^17^,^18^.

The extraordinary breadth and potency of some of the newer bnAbs also afford promising opportunities for immunotherapy of established infection. Recent proof-of-concept studies with passively delivered bnAbs in HIV-infected humanized mice and simian–human immunodeficiency virus (SHIV)-infected macaques have generated encouraging therapeutic results, especially when combinations of bnAbs were used^19^,^20^,^21^,^22^,^23^. Moreover, a single infusion with the CD4bs bnAb, 3BNC117, was recently shown to reduce plasma viral load by 0.8–2.5 log_10_ in chronically infected humans^24^. These therapeutic benefits might be improved in the presence of standard antiretroviral drugs^20^ and host autologous neutralizing antibodies^21^.

Measurements of bnAb potency and breadth are traditionally determined by the concentration of antibody that inhibits either 50% (IC_50_) or 80% (IC_80_) of a fixed virus inoculum in a dose–response single-cycle infection assay *in vitro*. While these neutralization thresholds might be sufficient in a prophylactic vaccine setting, where the multiplicity of infection during transmission is relatively low^25^,^26^,^27^ they fall far below the effective therapeutic dose range that will be required to inhibit multiple logs of virus and impede escape in an infected individual. Another clinically relevant dimension of dose–response curves is slope, which may be a more accurate measure of potency at therapeutically relevant inhibition levels. Studies with antiretroviral drugs have shown that the slope can be used to more reliably predict clinical outcome than IC_50_ alone. Instantaneous inhibitory potential (IIP) is an additional pharmacodynamic metric that goes further by incorporating both slope and IC_50_ to predict the number of logs of infection reduced at any given concentration of drug in a single-round infection assay^28^,^29^. Together, IC_50_ and slope determine the full range of activity for a given antiretroviral agent, and IIP puts these parameters into a more clinical context. For antiretroviral drugs, the slope of the sigmoidal dose–response curve is related to specific inhibitory mechanisms defined by the cooperative reactivity of inhibitors and their targets^28^,^30^,^31^,^32^.

Here we show that the neutralization slopes of bnAbs play an important role in forming therapeutic expectations from *in vitro* neutralization curves and can complement and extend traditional IC_50_/IC_80_-based analyses. We also find that slope is more strongly associated with neutralization breadth than IC_50_. With some exceptions, bnAb slopes generally segregate by epitope class suggesting that like HIV inhibitors, bnAb slopes are also related to specific mechanisms of neutralization, thus, this parameter might aid in the development of novel, highly effective immunotherapies. While both slope and IC_50_ are fundamental properties of bnAb activity *in vitro*, bnAb slopes are rarely considered when predicting therapeutic potency. Our results show that this mechanistic parameter has a significant impact on predicted therapeutic potency and adds a new dimension to the development of novel immunotherapeutics.

## Results

### Impact of slope on predicted therapeutic potencies of bnAbs

IC_50_ and IC_80_ are common metrics used to establish clinical expectations of bnAb activity from experimental results *in vitro* and to identify bnAbs with high potential for advancement into clinical trials. While useful, these parameters alone offer only a limited description of neutralization activity. An additional and often neglected parameter, the dose–response slope, was strongly associated with clinical outcome in the context of small-molecule HIV inhibitors, which exhibited a wide range of class-specific and mechanism-specific slopes^29^,^30^,^31^,^32^. To our knowledge, only one previous study examined in any detail the slopes of HIV-1 bnAb dose–response curves, and this was mostly done in the context of assessing the effects of combinations with earlier bnAbs: b12, 2G12 and 2F5 (ref. 33). Here we obtained dose–response curve slopes for 14 bnAbs and soluble CD4 (sCD4) assayed in TZM-bl cells against a global panel of 12 molecularly cloned HIV Env-pseudotyped reference viruses^34^ (Supplementary Table 1). To acquire additional positive neutralization results, a subset of bnAbs was assayed against five additional Env-pseudotyped reference viruses^35^ (Supplementary Table 1). The bnAbs represented six epitope classes including the CD4bs bnAbs VRC01 (refs 1, 4), 3BNC117 (ref. 3), CH31 (ref. 4) and HJ16 (ref. 2); the V2-glycan bnAbs PG9, PG16 (ref. 5) and CH01 (ref. 8); the V3-glycan bnAbs PGT128 (ref. 6), 10-1074 (ref. 7) and PGT121 (ref. 6); the high mannose cluster (HM cluster) bnAb 2G12 (ref. 36); the gp41 MPER bnAbs 2F5, 4E10 (refs 10, 11) and 10E8 (ref. 9); and the gp120/gp41 glycan bnAb PGT151 (ref. 14).

**Figure 1:**
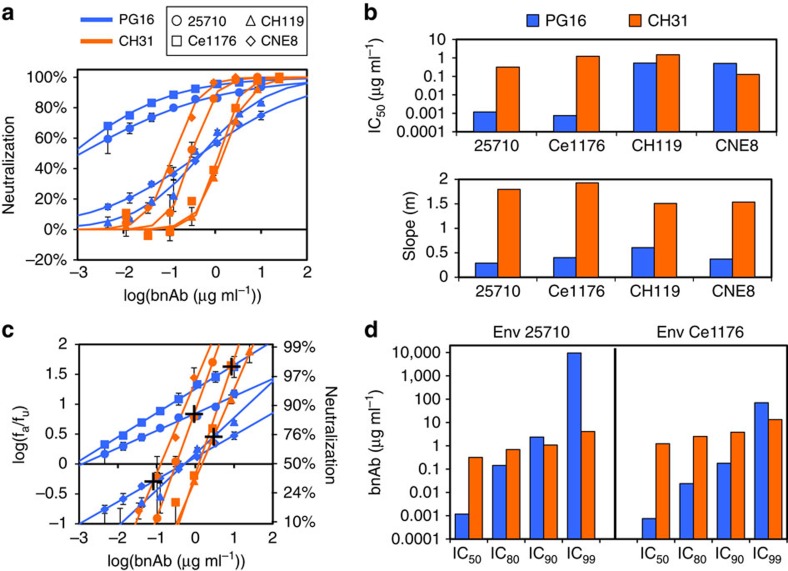
Effect of the slope on neutralization and potency. (**a**) Hill plots of neutralization curves for PG16 (blue) and CH31 (orange) against four representative Envs from our panel. (**b**) IC_50_ (top) and slope (bottom) values determined by median-effect fitting (Methods). (**c**) Linear median-effect plots of neutralization for the same data in **a**, where IC_50_ falls at the *x* intercept and slope describes the angle of each curve relative to the *x* axis. Intersections (crosses) indicate where both PG16 and CH31 gave the same neutralization at the same concentration for each Env. (**d**) Potencies of PG16 and CH31 against two Envs with the greatest difference in IC_50_. Data shown in (**a**) and (**c**) are the average of two replicates and error bars indicate s.d.

Dose–response neutralization curves for PG16 (V2 glycan) and CH31 (CD4bs) assayed against four Envs are shown in Fig. 1a as examples of some of the most marked slope differences observed. Regardless of differences in IC_50_ (Fig. 1b, top), PG16 exhibited a shallow dose-dependent rise in neutralization relative to the steeper rise seen with CH31 (Fig. 1a), which is indicated by the lower dose–response curve slope for PG16 (Fig. 1b, bottom; compare blue with orange bars). These results were transformed using the median-effect equation^37^ (equation (1), Supplementary Fig. 1, where *f*_a_ is percent neutralization, *D* is antibody concentration, *D*_m_ is IC_50_ and *m* is slope), to give the linear dose–responses shown in Fig. 1c. This form reveals that for any given Env, the higher slope of CH31 relative to PG16 causes the corresponding neutralization curves to converge towards an intersection point and then diverge as concentration continues to increase. This intersection defines the concentration (*D*_i_; equation (2), where *D*_m,1_, *m*_1_ and *D*_m,2_, *m*_2_ are the IC_50_s and slopes for PG16 and CH31, respectively) and inhibition level (*f*_ai_; equation (3), where *D*_m_ and *m* are the IC_50_ and slope of either PG16 or CH31, respectively) at which both PG16 and CH31 were equally effective against the same Env.




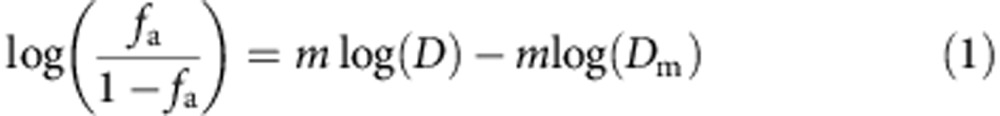







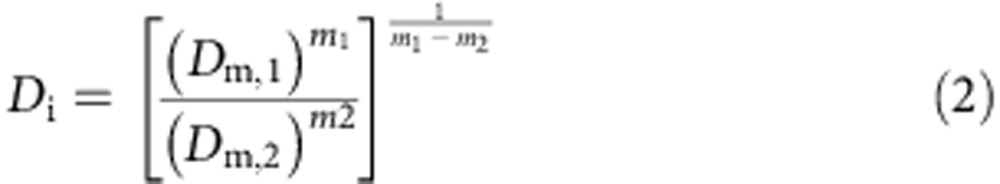







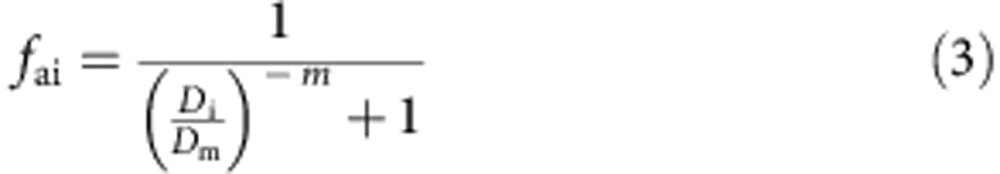




The impact of these intersections on potency is illustrated in Fig. 1d, where the 50% inhibitory concentration of PG16 was 250- and 1,500-fold lower than CH31 for Envs 25710 and Ce1176, respectively. The potency of CH31 progressively approached that of PG16 for 80 and 90% inhibition, where eventually CH31 was 2,000- and 5-fold more potent than PG16 for 99% inhibition, reflecting the convergence, intersection and divergence of the curves. Median-effect extrapolations to ≥99% inhibition were experimentally verified using a titre-reduction assay, where CH31 produced a 3-log reduction in viral titre at 10 times its IC_80_ concentration (42 μg ml^−1^) compared with PG16, which produced only a 1.9-log reduction in viral titre at 10 times its IC_80_ (0.44 μg ml^−1^) against Env Ce1176 (Supplementary Fig. 2). Thus, changes in relative potency at therapeutically relevant bnAb concentrations are the direct result of the slope’s differential effect on neutralization. Importantly, these same slope-driven features were strongly associated with the clinical activity of HIV inhibitors, while IC_50_ alone was not correlated to the historical clinical properties of HIV inhibitors^28^,^30^.

### BnAb classes have characteristic slopes

**Figure 2:**
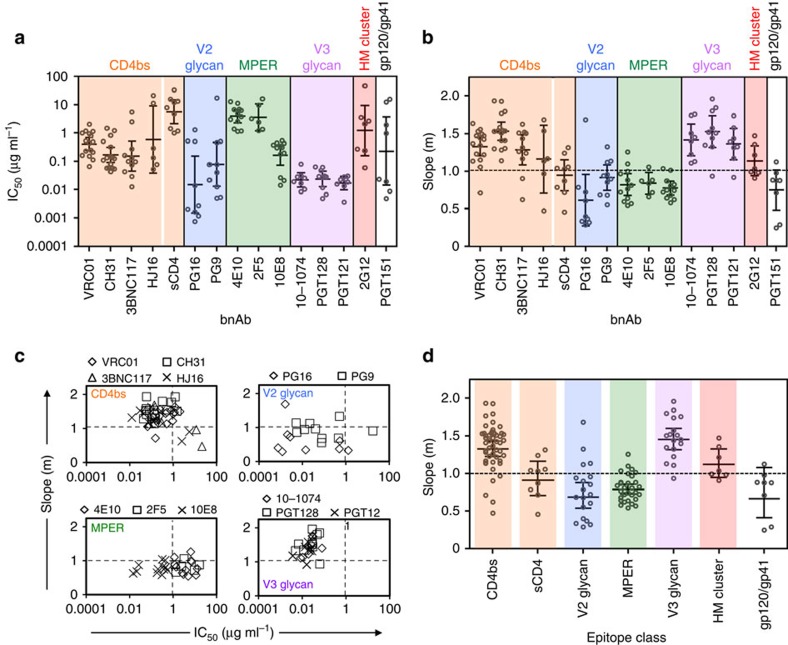
Slope and IC_50_ characteristics of bnAb epitope classes. (**a**) IC_50_ and (**b**) slope values of bnAbs and sCD4 (bottom categories) against each Env in our panel (circles). Bars indicate geometric mean IC_50_s with 95% confidence interval (CI) or mean slopes with 95% CI. Antibodies are grouped by epitope class (top categories). (**c**) Landscape of slope and IC_50_ values for CD4bs (top left), V2-glycan (top right), MPER (bottom left) and V3-glycan (bottom right) bnAb classes. Dashed lines indicate quadrants of high/low IC_50_ and high/low slope. (**d**) Slopes of each bnAb epitope class against each Env in our panel. Bars indicate mean and 95% CI. All data shown are from bnAb/Env combinations that achieved complete neutralization and are derived from median-effect fits of two-replicate averages.

The IC_50_ and slope values for each bnAb assayed against our entire panel of Envs (Supplementary Table 1) are shown in Fig. 2a,b (see also Supplementary Table 2), illustrating the full range of values observed in the complete data set. Virus/bnAb combinations that did not reach at least 50% neutralization at the highest bnAb concentrations tested were excluded due to weak or non-detectable activity. We also note that some neutralization curves exceeded 50% but plateaued below 100% (Supplementary Fig. 3), indicating that a portion of the virus was refractory to the bnAb. Consistent with previous reports^5^,^13^,^15^,^38^,^39^ we mostly observed such incomplete neutralization for glycan-targeting bnAbs (CH01, PG16, PG9, 2G12 and PGT151). Incomplete neutralization of genetically clonal Env-pseudovirions is likely a manifestation of alternative post-translational modifications giving rise to a heterogenous population of Env spikes, resulting in an epigenetic mixture of sensitive and resistant virions. Examples are post-translational variability in sequon occupancy^40^ and glycan composition^38^,^41^, both of which could profoundly affect bnAbs that either require glycan as part of their epitope, or are subject to glycan shielding. BnAbs that are better able to tolerate this epigenetic variability are more likely to achieve 100% neutralization in the assay. We excluded bnAb/Env combinations that exhibited incomplete neutralization (that is, curves that plateau below 95%) because their full neutralization potential fell within the measurable range of the assay (<1-log reduction in infectivity). To compensate for minor assay variance, 95% was used as the upper threshold for plateaus that were considered truly indicative of incomplete neutralization. CH01 exhibited plateaus below 95% neutralization against every Env in our panel, while such plateaus for PG16, PG9, 2G12 and PGT151 were only observed among a minor subset of 1–2 Envs (Supplementary Table 2).

Collectively, few statistically significant differences in slope were observed within each bnAb epitope class for those bnAb/Env combinations achieving complete neutralization within our detection limits, suggesting that slope is primarily a feature of the target epitope. One exception to this general rule was sCD4, which gave slopes significantly lower than those of the CD4bs bnAb class (0.95±0.3 for sCD4 and 1.37±0.3 for CD4bs bnAbs combined, *P*<0.001, Student’s *t*-test). Although the slopes of PG9 were generally higher than those of PG16, this difference did not reach statistical significance (0.92±0.2 for PG9 and 0.61±0.4 for PG16, *P*=0.08). No significant correlation between slope and IC_50_ was observed for any bnAb class, reflecting the fundamental independence of these two parameters. However, each class of bnAbs clustered differentially in the landscape of IC_50_ and slope values (Fig. 2c). That CD4bs (high slope/moderate IC_50_, excluding sCD4), V2-glycan (low slope/dispersed IC_50_), MPER (low slope/high IC_50_) and V3-glycan (high slope/low IC_50_) bnAbs clustered into distinct quadrants suggest that bnAbs in each particular class occupy a different phenotypic landscape defined by both IC_50_ and slope. The 10E8 MPER bnAb represents another interesting exception as it exhibited significantly lower IC_50_s than 4E10 and 2F5 despite having similar slopes (geometric mean IC_50_ for 10E8=0.16 μg ml^−1^ versus 3.5 and 3.6 μg ml^−1^ for 4E10 and 2F5, respectively; *P*<0.01, one-way analysis of variance (ANOVA); Fig. 2a,b).

The slopes of each bnAb epitope class could be further categorized into the three groups (Fig. 2d) as those having slopes >1 (CD4bs, V3 glycan), those having slopes ∼1 (HM cluster) and those having slopes <1 (V2 glycan, gp120/gp41, MPER) with high statistical significance (*P*<0.0001, one-way ANOVA). Notably, sCD4 and each bnAb exhibited a range of slope values among the viruses in our panel, indicating that the slope is also Env dependent. This will be an important consideration when interpreting clinical benefits among a patient population receiving passive bnAb therapy.

### CD4-based immunoadhesins

In addition to *bona fide* bnAbs, immunoadhesins consisting of effector domains fused to the IgG Fc region represent a novel class of rationally designed antiviral therapeutics. For example, CD4-Ig consists of the CD4 D1 and D2 domains fused to the Fc domain of IgG1 (IgG1-Fc). Very recently, an enhanced version (eCD4-Ig) was described in which a mimetic peptide derived from the N terminus of the major HIV coreceptor, CCR5, was fused to the C terminus of the CD4-Ig Fc domain^42^. eCD4-Ig was able to neutralize an exceptionally broad array of HIV-1 Envs with an increased potency relative to CD4-Ig. We analysed the dose–response curves of eCD4-Ig and CD4-Ig to determine if differences in slope may account for the enhanced potency of eCD4-Ig and compared these with the slopes and IC_50_s of the CD4bs bnAbs and sCD4. First-order approximations of slopes can be obtained from available IC_50_ and IC_80_ concentrations by using the linear median-effect form (see equation (6) in Methods), thus, median effect reduces the complex curvature of the standard sigmoidal Hill curve into a linear form (Fig. 1c and Supplementary Fig. 1) that simplifies mathematical analysis and allows one to approximate slopes from a limited set of available data^37^. Both IC_50_ and IC_80_ values are publicly available for the CD4-based immunoadhesins.

**Figure 3:**
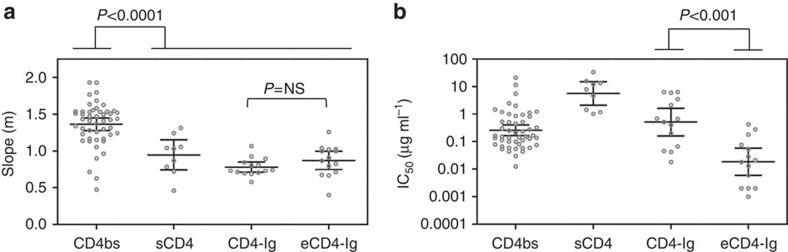
Slope and IC_50_ characteristics of CD4 immunoadhesins. Neutralization slopes (**a**) and IC_50_s (**b**) of the CD4bs bnAbs combined and CD4 immunoadhesin reagents sCD4, CD4-Ig and eCD4-Ig. Slope and IC_50_ values for CD4bs bnAbs and sCD4 are from Fig. 2 and reproduced here for ease of comparison. Slopes for CD4-Ig and eCD4-Ig were estimated from published IC_50_ and IC_80_ values^42^ using equation (6) (Methods). Thus, slopes were only estimated for a subset of Envs (*n*=14) where discrete IC_50_ and IC_80_ values were reported. Bars represent geometric mean IC_50_ with 95% CI or mean slope with 95% CI and *P* value (one-way ANOVA) compares slopes of CD4bs bnAbs with sCD4, CD4-Ig and eCD4-Ig combined in panel **a**, all other *P* values are Student's *t*-test.

Figure 3a,b shows the approximated slopes and published IC_50_ values for CD4-Ig and eCD4-Ig. Interestingly, Fig. 3a shows that eCD4-Ig does not have a consistently higher slope compared with CD4-Ig across the panel of Envs analysed (0.78±0.12 and 0.87±0.21 for CD4-Ig and eCD4-Ig, respectively). Indeed, both the CD4 immunoadhesins and sCD4 all have similar slopes (0.95±0.27 for sCD4). However, eCD4-Ig consistently exhibited a 1.4 log lower IC_50_ compared with CD4-Ig (*P*<0.001, Student’s *t*-test) (Fig. 3b). Thus, the enhanced potency of eCD4-Ig relative to CD4-Ig can be attributed to its lower IC_50_. That the slope does not differ between sCD4, CD4-Ig and eCD4-Ig may also indicate that these mechanisms of inhibition are the same and are predominated by the initial CD4-binding event. Conversely, the higher slopes of CD4bs bnAbs relative to sCD4, CD4-Ig and eCD4-Ig suggest the neutralizing mechanisms of these bnAbs might be distinct from those of the CD4 immunoadhesins.

### Neutralization breadth is strongly associated with slope

**Figure 4:**
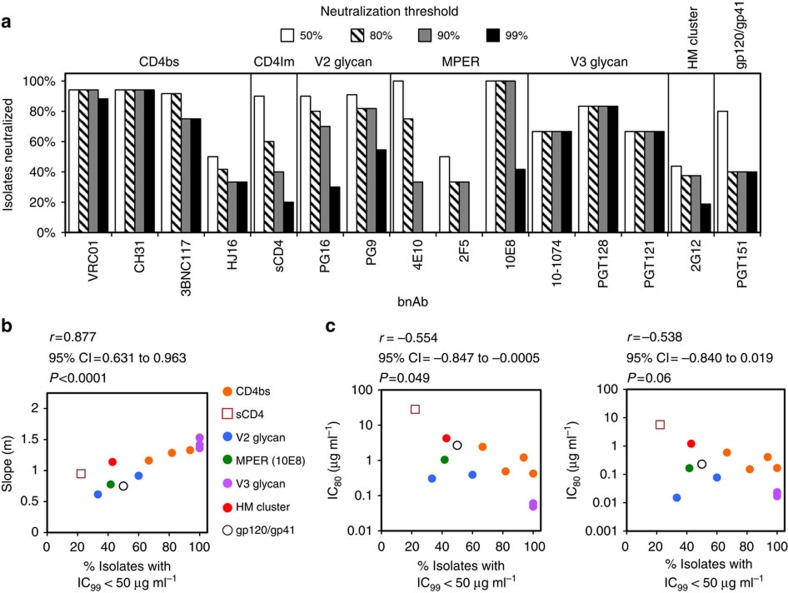
Effect of slope on neutralization breadth. (**a**) Neutralization breadths determined from dose–response curves of each bnAb against our total Env panel at increasing neutralization thresholds. A bnAb is considered non-neutralizing for a particular Env at a given inhibitory threshold when the respective inhibitory concentration (IC_50_, IC_80_, IC_90_ or IC_99_) is >50 μg ml^−1^. Antibodies are ordered by epitope class. Correlations of slope (**b**), IC_80_ (**c**, left) and IC_50_ (**c**, right) to breadth at 99% neutralization for each bnAb, grouped by epitope class (symbols), where breadth excludes Envs giving no detectible neutralization within the parameters of our assay (Methods). Pearson correlations (*r*), 95% CI and associated *P* values are indicated above each graph.

The overall therapeutic potential of bnAbs will depend on the diversity of HIV isolates that are neutralized within a clinically relevant range of concentration. Breadth is traditionally defined as the percentage of isolates for which a bnAb can achieve 50 or 80% neutralization below a designated concentration, usually 10–50 μg ml^−1^. Because slope defines the changes in bnAb concentration necessary to increase inhibition, we sought to determine how this property affects neutralization breadth at increasing therapeutic thresholds, rather than simply using IC_50_ and IC_80_ values at a fixed bnAb concentration. Figure 4a shows the breadth of each bnAb at increasing thresholds of IC_50_, IC_80_, IC_90_ and IC_99_ using median-effect-fitted curves. Breadth scores across these increasing thresholds changed more markedly for bnAbs with characteristically lower slopes (V2 glycan, MPER and gp120/gp41) than for bnAbs with characteristically higher slopes (CD4bs and V3 glycan). The improved and narrow distribution of potencies for 10E8 resulted in a delay of this effect to higher neutralization thresholds, where the extrapolated IC_99_ breadth decreases from 100 to 40%. For isolates that were sensitive to each bnAb (IC_50_ <50 μg ml^−1^), breadth at the more therapeutically relevant IC_99_ threshold (that is, potency needed for 2-log inhibition) was strongly associated with slope (Fig. 4b). Traditional measures of potency showed moderate association with neutralization breadth (IC_80_) or none at all (IC_50_) (Fig. 4c). The MPER bnAbs 4E10 and 2F5, with the exception of 10E8, were excluded from this latter analysis because they exhibited zero breadth at the IC_99_ threshold.

### IIP defines clinical expectations using both IC_50_ and slope

The neutralization potency of an antibody can be more completely described when both slope and IC_50_ are used to determine the IIP. IIP was first used in an explanatory framework that accounted for the marked differences in clinical potency among the extant classes of antiretroviral drugs^28^,^29^, which could not be accounted for by differences in IC_50_ alone. IIP uses both slope and IC_50_ to describe the log decrease by which single-round infection is reduced by the antiviral agent at a given concentration (*D*) *in vitro* (see equation (4), where slope is *m* and IC_50_ is *D*_m_, and Supplementary Fig. 4). Thus, IIP serves as a more precise therapeutic expectation for any given dose of bnAb than traditional metrics (IC_50_ and IC_80_).




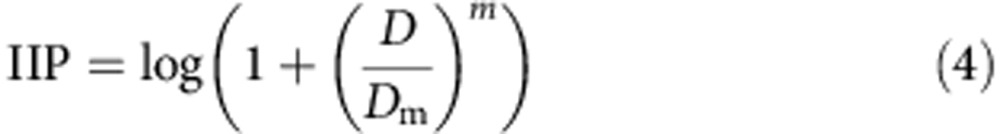




**Figure 5:**
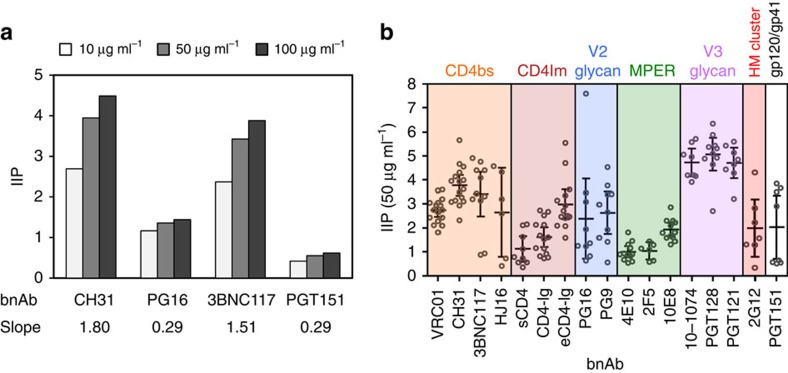
IIP incorporates both slope and IC_50_. (**a**) The 10, 50 and 100 μg ml^−1^ IIPs for CH31, PG16, 3BNC117 and PGT151 against Env 25710 with slopes indicated. (**b**) 50 μg ml^−1^ IIPs for all bnAbs against our Env panel where complete neutralization was observed. Bars indicate mean and 95% CI.

As an exponential parameter, small differences in slope (*m*) can lead to large differences in IIP as *D* increases. As an illustrative example, we calculated the IIP of four bnAbs against a single Env clone (25710) at 10, 50 and 100 μg ml^−1^ (Fig. 5a). These four bnAbs from the indicated epitope classes (CD4bs, V2 glycan and gp120/gp41) also exhibit different *m* values with this Env. A marked increase in IIP (on a log-scale) is seen as the concentration of bnAb increases, most notably for bnAbs with higher slopes (CH31 and 3BNC117). This pattern is even more apparent when examining the dose–response curves shown in Supplementary Fig. 4.

Using equation (4) and the same methodology, we calculated the IIP of the entire panel of bnAbs at 50 μg ml^−1^, which is a common threshold concentration used to assess the therapeutic potential of bnAbs (Fig. 5b). By incorporating both slope and IC_50_, IIP reveals some striking results. For example, the V3-glycan bnAbs with higher slopes (1.5±0.3) were predicted to reduce viral infectivity by 3 logs more than the MPER bnAbs with lower slopes (0.8±0.2) (mean IIP 4.9±0.9 for V3-glycan bnAbs versus IIP 1.4±0.6 for MPER bnAbs; *P*<0.0001, Student’s t-test). In general, the IIP reflected the slopes (Fig. 2b) of each bnAb class except the V2-glycan bnAbs, where the wide range of IC_50_ values (Fig. 2a) resulted in an equally wide distribution of IIPs. Indeed, PG16 exhibited one of the highest IIPs (7.6) against Env 703010217, which reflected the characteristically low IC_50_ (0.002 μg ml^−1^) of PG16 when coupled to an unusually high slope (1.69) for this bnAb (median PG16 slope 0.6±0.4) against this particular Env. The IIPs of the CD4 immunoadhesin reagents (CD4Im) reflected their differences in IC_50_, where eCD4-Ig achieved a 1.4 log greater reduction in infection than CD4-Ig (IIP=1.6±0.7 for CD4-Ig and 3.0±1.0 for eCD4-Ig, *P*<0.001). Recall that the IC_50_s of eCD4-Ig were 1.4 logs lower than those for CD4-Ig but no significant differences in slope were observed (Fig. 3). The same was observed for 10E8, which gave geometric mean IC_50_s that were 1.3 logs lower than 2F5 and 4E10 resulting in ∼1-log increase in IIP. Altogether, these data suggest that the combination of slope and IC_50_ values reflected in the IIP metric has considerable explanatory potential that can complement and inform evaluation of the therapeutic efficacy of bnAbs in the same way these three metrics illuminate our understanding of the clinical potency of small-molecule inhibitors.

### Clinical implications of bnAb slopes

**Figure 6:**
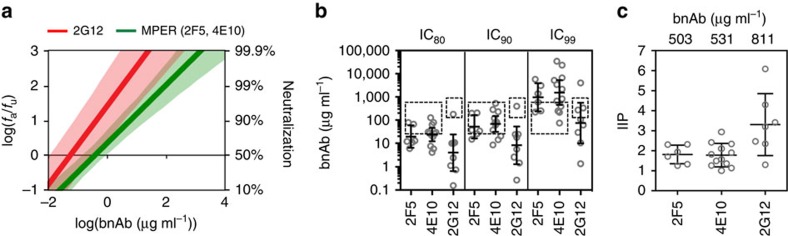
Sensitivity of therapeutically relevant potencies to small differences in slope. (**a**) Median-effect plot of mean neutralization for 2G12 (red solid line) and MPER bnAbs (4E10 and 2F5 combined, green solid line) determined from the combined median-effect curves of these bnAbs against each Env on our panel that was neutralized. Shaded areas indicate the corresponding s.d. (**b**) IC_80_, IC_90_ and IC_99_ potencies of 2F5, 4E10 and 2G12 against each Env in our panel (symbols). Bars indicate geometric mean and 95% CI. Dotted boxes illustrate the range of peak/trough plasma concentrations for each bnAb reported in human trials^38^,^39^. (**c**) IIPs of 2F5, 4E10 and 2G12 against our Env panel at average peak serum concentrations reported in human trials^43^,^44^.

Two studies in humans demonstrated a moderate transient reduction in plasma viraemia when 2G12, 2F5 and 4E10 were co-administered immediately before treatment interruption in subjects who began standard antiretroviral therapy during acute infection^43^,^44^. Results of a detailed analysis of escape variants in the treated subjects suggested that 2G12 was the only antibody in the combination that exerted pressure on the virus. On the other hand, results of an in-depth analysis showing that escape *in vitro* may be more difficult for 2F5 and 4E10 than for 2G12 suggests that perhaps all three bnAbs were needed for the observed transient effect on viraemia^45^. We observed in our data set moderate but statistically significant differences in slope between 2G12 and the MPER bnAbs 2F5 and 4E10 (2G12 slope=1.1±0.2; combined 2F5 and 4E10 slopes=0.83±0.2, *P*=0.002, Student’s *t*-test). As shown in Fig. 6a, this moderate difference is compounded at higher neutralization thresholds, such that 2G12 achieves 99% neutralization at an average of 75 μg ml^−1^, whereas 2F5 and 4E10 required ≥1 mg ml^−1^. Figure 6b shows the range of 2F5, 4E10 and 2G12 peak/trough plasma concentrations estimated from human trials^43^,^44^. While the IC_80_s and IC_90_s of 2F5 and 4E10 fall within or below this range, only 2G12 remained predominantly within or below this range at IC_99_. The IIP of these bnAbs against our Env panel at average peak serum concentrations^43^,^44^ provide a more clinical description of expected efficacy, where 2F5 and 4E10 achieved a narrow distribution of moderate IIP (1.64±0.44 and 1.68±0.56 for 2F5 and 4E10, respectively) and 2G12 achieved an IIP >3 for over half the Envs on our panel that were sensitive to this bnAb (Fig. 6c, mean IIP 3.3±1.5). These results illuminate potential mechanisms for the exclusive 2G12 escape observed with this triple therapy in humans, in addition to the relative ease of 2G12 escape *in vitro*^46^ and the distinct pharmacokinetic properties of these three bnAbs, where accumulation of 2G12 results in greater concentrations *in vivo*^43^,^44^. The higher average IIP of 2G12 against our Env panel suggests this bnAb would likely exert a greater neutralizing activity and selective pressure than 2F5 or 4E10; however, the broad distribution of 2G12 IIP relative to the narrow distribution of MPER IIP also suggest a broader landscape of potential resistance mutations for 2G12, represented by our Env panel. Overall, our results suggest that even subtle differences in slope can give rise to important differences in IIP and clinical outcome.

Three new bnAbs have been evaluated in passive immunotherapy experiments in macaques, each of which exhibited characteristic slopes >1 in our study. As monotherapy, PGT121 (V3 glycan) was profoundly effective against established SHIV-SF162P3 infection^23^, whereas 3BNC117 (CD4bs) and 10-1074 (V3 glycan) were profoundly effective against established SHIV-AD8EO infection^22^, resulting in up to 3-log reductions in plasma viraemia in each case. Using available published dose–response data, we estimated the slope for PGT121 against the SHIV-SF163P3 challenge stock to be ∼2, and the slopes for 3BNC117 and 10-1074 against SHIV-AD8EO challenge stock to be 1.59 and 1.90, respectively. Finally, as mentioned earlier, a single infusion with 3BNC117 was recently shown to reduce plasma viral load in chronically infected humans as long as therapeutic levels were present^24^. While these results further indicate that bnAbs with slopes >1 are associated with positive clinical outcomes, a paucity of passive immunotherapy data with bnAbs that exhibit lower slopes precludes quantitative verification of their potential clinical benefits at this time.

## Discussion

Next-generation bnAbs are currently being considered for immunotherapy due to their enhanced potency and breadth of neutralization. In addition to these factors, other considerations such as scale-up manufacturability, safety, pharmacokinetics, immunogenicity and ease of escape, just to name a few, will determine the clinical success of bnAbs. Furthermore, neither breadth nor potency (or any *in vitro* test for that matter) can easily predict the ease of escape and fitness of escape mutations to any particular bnAb *in vivo*. Indeed, it is unlikely that monotherapy with any one bnAb, no matter how potent or broad, will succeed, especially since all extant bnAbs have known resistance mutations.

Nonetheless, breadth and potency, imperfect surrogate measures as they are of therapeutic efficacy, are critical components of the evaluation of bnAb candidates (or bnAb combinations) for *in vivo* efficacy trials. Importantly, breadth and potency are traditionally defined by *in vitro* IC_50_ and IC_80_ values that are well below the therapeutic threshold and these metrics only offer a limited, fixed description of bnAb activity. Here we show that the dose–response curve slope is a more reliable indicator of bnAb breadth and potency at more therapeutically relevant doses. The current state-of-the-art does not consider the slope parameter when prioritizing which bnAb or combination of bnAbs to advance to human trials. We believe our analysis can complement these increasingly sophisticated efforts^16^ and enhance the clinically predictive power of *in vitro* surrogate assays for bnAb potency.

More importantly, inclusion of the established IIP metric, which incorporates both IC_50_ and slope, adds another clinically relevant dimension to our analysis (Fig. 5). The IIP metric has proven utility in predicting the clinical potency of antiretroviral drugs and drug combinations^28^,^29^,^32^. It is derived from a pharmacodynamic model that predicts the log decrease in virus infection when the antiviral agent, in this case the bnAb, is extrapolated to a given clinically relevant concentration. Predicted IIPs or IIP_ave_ (a more sophisticated metric that includes additional pharmacokinetic parameters such as half-life of the bnAb, but critically still includes the IC_50_ and slope parameters) can help guide the determination of effective dosing ranges and intervals.

Mechanistic explanations for the different bnAb curve slopes will require additional studies. We hypothesize that for the genetically cloned Env-pseudovirions used here, slope is at least partially determined by epigenetic heterogeneity within the Env glycoprotein spikes that decorate the virus surface. Examples are variability in sequon occupancy and glycan composition as mentioned above. Target heterogeneity has been invoked to explain the slopes of other ligand-effector units^47^ and was suggested to impact the slope of HIV inhibitors, including bnAbs such as b12, 2G12 and 2F5 (ref. 33). BnAbs that are better able to tolerate post-translational Env heterogeneity, or whose epitopes are not affected by this, would neutralize all virus particles equally well, resulting in a slope of ∼1. BnAbs with a lower threshold of tolerance would exhibit variable neutralization efficiencies across the heterogeneous virus population, resulting in slopes <1. Here adequate bnAb concentrations may be capable of neutralizing all virions in the population; however, it is also possible that a minor fraction of virions would completely resist neutralization. Because we excluded all neutralization curves that exhibit incomplete neutralization in our assay, the expected plateau representing the minor fraction of resistant virions would reside outside the range of the assay (for example, plateau at 99.9% neutralization). Neutralization assays with a wider range of detection will be needed to assess this latter possibility. BnAb slopes might also be determined in part by mechanisms of neutralization, such as an ability to act at one or multiple stages of the fusion process^48^,^49^. BnAbs that are able to inhibit at multiple stages might cooperate to explain in part dose–response curve slopes >1.

Collectively our data reveal an association between bnAb epitopes and dose–response slopes that bridge the fields of structural biology and clinical evaluation, and may help to guide the rational design and testing of therapeutically effective antibodies for HIV and other pathogens.

## Methods

### Virus stocks

Virus stocks were prepared by transfection in 293T cells and titrated in TZM-bl cells as described^50^. A complete list of our HIV Env panel is provided in Supplementary Table 1.

### Neutralization assay

The neutralizing activity of bnAbs was measured as a function of reductions in luciferase (Luc) reporter gene expression after a single round of infection in TZM-bl cells^50^. TZM-bl cells (also called JC57BL-13) were obtained from the NIH AIDS Research and Reference Reagent Program, as contributed by John Kappes and Xiaoyun Wu. This is a HeLa cell clone that was engineered to express CD4 and CCR5 (ref. 51) and to contain integrated reporter genes for firefly luciferase and *Escherichia coli* beta-galactosidase under control of an HIV-1 long terminal repeat^52^. Briefly, a pre-titrated dose of virus was incubated with serial threefold dilutions of test sample in duplicate in a total volume of 150 μl for 1 h at 37 °C in 96-well flat-bottom culture plates. Freshly trypsinized cells (10,000 cells in 100 μl of growth medium containing 75 μg ml^−1^ diethylaminoethyl dextran) were added to each well. One set of eight control wells received cells+virus (virus control) and another set received cells only (background control). After 48 h of incubation, 100 μl of cells was transferred to a 96-well black solid plate (Costar) for measurements of luminescence using the Britelite Luminescence Reporter Gene Assay System (PerkinElmer Life Sciences). Assay stocks of molecularly cloned Env-pseudotyped viruses were prepared by transfection in 293T/17 cells (American Type Culture Collection) and titrated in TZM-bl cells as described^50^. This assay has been formally optimized and validated^53^ and was performed in compliance with good clinical laboratory practices, including participation in a formal proficiency testing programme^54^. Additional information on the assay and all supporting protocols may be found at: http://www.hiv.lanl.gov/content/nab-reference-strains/html/home.htm.

### BnAbs

3BNC117 (ref. 3) and 10-1074 (ref. 7) were obtained from Michel Nussenzweig (Rockefeller University). VRC01 (refs 1, 4) was obtained from John Mascola (Vaccine Research Center, NIAID, NIH). PG9 (ref. 5), PG16 (ref. 5), PGT128 (ref. 6) and PGT151 (ref. 14) were obtained from Dennis Burton (Scripps Research Institute). CH01 (ref. 8) and CH31 (ref. 4) were obtained from Barton Haynes (Duke University Medical Center). HJ16 (ref. 2) was obtained from Davide Corti and Antonio Lanzavecchia (Institute for Research in Biomedicine, USI Switzerland). 2G12 (ref. 36), 2F5 (refs 10, 11) and 4E10 (refs 10, 11) were purchased from PolyMun Scientific (Germany).

### Median-effect analysis

Slope and IC_50_ values were determined using the median-effect method^37^. This method involves a linear transformation of the standard Hill plot (Supplementary Fig. 1a), where neutralization is represented by a log effect ratio (equation (1) and Supplementary Fig. 1b). Linear regression was used to determine the slope (*m*) and IC_50_ (*D*_m_) values corresponding to the linear slope and *x* intercept, respectively, of each curve (Supplementary Fig. 1b). In all cases, median-effect fits were determined from the average of two experimental replicates for each neutralization curve. Envs that did not reach a minimum 50% neutralization within the range of antibody concentrations used in each neutralization assay were considered non-neutralized as well as Envs with IC_50_ values above 50 μg ml^−1^ (Supplementary Table 2). Neutralization intersection concentrations (*D*_i_) were determined using equation (2), which was derived from equation (1). In cases where neutralization reached a maximum plateau <95% (Supplementary Table 2 and Supplementary Fig. 3) a stepwise iterator (perl v5.12.4) was used to fit maximum neutralization (*N*) to equation (5) using the method of least squares, where *f*_a_ is neutralization as a percentage of maximum neutralization, *N* is maximum neutralization, *D* is bnAb concentration (μg ml^−1^), *m* is slope and *D*_m_ is the concentration giving half maximum neutralization.




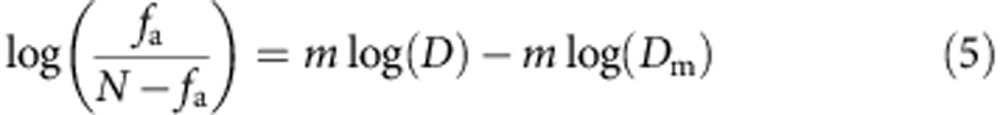




In all cases, the IC_50_ values reported are the concentrations giving 50% maximum neutralization.

### IIP analysis

IIPs were calculated using equation (4) as previously described^29^, using fitted slope (*m*) and IC_50_ (*D*_m_) values. See Supplementary Fig. 4 for an illustrative description of IIP.

### Slope estimates

Equation (6) was used to estimate slope values from publically available IC_50_ and IC_80_ values, where *m* is slope and log(4) is the change in the log effect ratio (
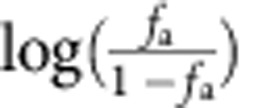
, equation (1)) between 50 and 80% neutralization. Equation (6) was derived from the linear median-effect form described by equation (1).




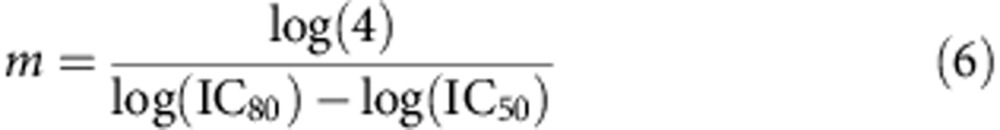




### Comparison of epitope classes

Statistical differences in mean slope between the CD4bs, V2 glycan, V3 glycan, MPER, HM cluster or gp120/gp41 classes were performed using one-way ANOVA in GraphPad Prism 6. The variance of slopes among these classes were equal and followed a normal distribution.

### Experimental validation of extrapolated potencies

Extrapolation of inhibitory concentrations using median-effect-fitted slope and IC_50_ values was investigated experimentally using a modification of the TZM-bl assay described above. Briefly, undiluted stocks of Env-pseudotyped viruses were incubated in the presence and absence of the indicated concentrations of bnAbs for 1 h at 37 °C. Each mixture was then diluted serially fourfold in quadruplicate for a total of 12 dilutions in 96-well culture plates. TZM-bl cells were added and incubated at 37 °C for 48 h. Infectious viral titre was defined by the fold dilution of Ce1176 virus stock, virus stock+CH31 or virus stock+PG16 mixtures giving 1,000 relative light units (RLU) luciferase activity. The dynamic range of this assay was greater than the standard TZM-bl neutralization assay, where we observed a maximum 3.2-log reduction in virus titre (∼99.9% neutralization). The change in virus titre reduction between 2 × and 10 × IC_80_ concentrations for PG16 and CH31 were proportionate to their respective slopes, where the log titre reduction was 2.9-fold higher for CH31 (between 2 × and 10 × IC_80_) and the log titre reduction was 1.3-fold for PG16 (between 2 × and 10 × IC_80_) (Supplementary Fig. 2).

### Neutralization breadth and breadth correlations

Neutralization breadth was defined as the percentage of Envs on our panel that gave 50, 80, 90 or 99% neutralization at concentrations below 50 μg ml^−1^ according to median-effect fits. This calculation includes Envs for which no detectible neutralization could be experimentally observed. To accurately represent the correlations of slope, IC_50_ and IC_80_ to 99% neutralization breadth, breadths were re-calculated to exclude Envs that gave no detectible neutralization within the concentration range used in our assay.

### Statistical analysis

Slope and IC_50_ values were determined from linear regression of median-effect-transformed neutralization data using Microsoft Excel 2011. Pearson correlations, confidence intervals, *t*-tests and one-way ANOVA analyses were conducted using GraphPad Prism 6.

### Code availability

In cases where an Env/bnAb combination achieved a maximum plateau in neutralization within our detection limit, a least squares iterative algorithm was used to fit maximum neutralization (*N*) according to equation (5) using perl v5.12.4. This script is available as Supplementary Data 1.

## Additional information

**How to cite this article:** Webb, N. E. *et al*. Dose–response curve slope helps predict therapeutic potency and breadth of HIV broadly neutralizing antibodies. *Nat. Commun.* 6:8443 doi: 10.1038/ncomms9443 (2015).

## Supplementary Material

Supplementary Figures, Supplementary Tables and Supplementary References Supplementary Figures 1-4, Supplementary Tables 1-2 and Supplementary References

Supplementary Data 1 Script for the algorithm used to fit maximum neutralization according to Equation (5)
