# Supplementary material for: Dose–response curve slope helps predict therapeutic potency and breadth of HIV broadly neutralizing antibodies
Source: Nat Commun. 2015 Sep 29;6:8443. doi: 10.1038/ncomms9443 (PMC4588098; doi:10.1038/ncomms9443)
Supplement: Supplementary Figures, Supplementary Tables and Supplementary References — Supplementary Figures 1-4, Supplementary Tables 1-2 and Supplementary References [file ncomms9443-s1.pdf]

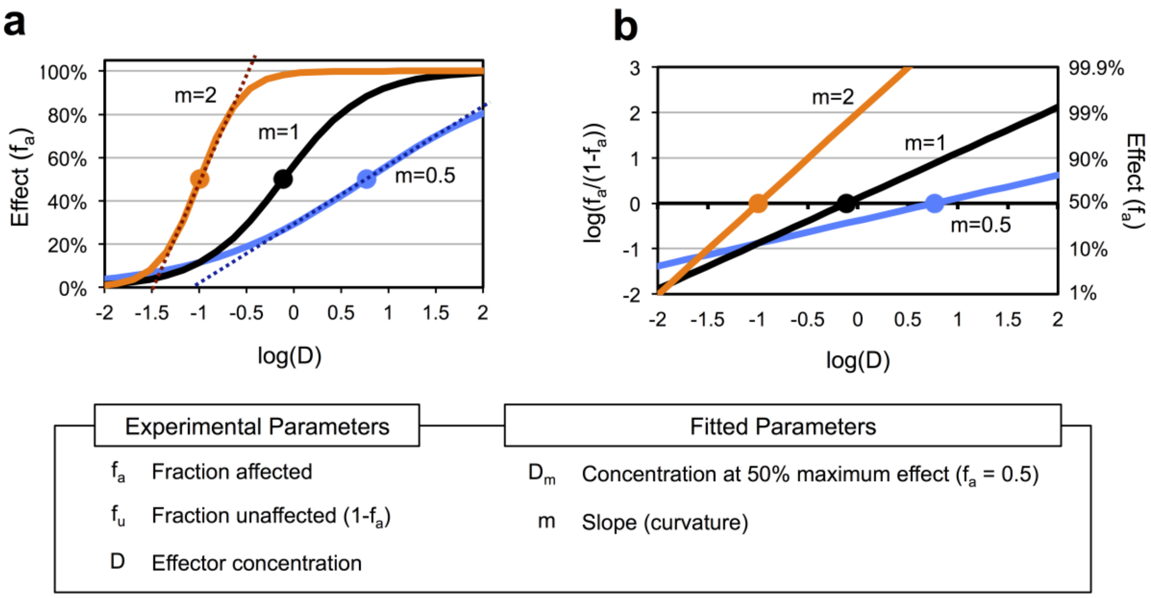

Supplementary Figure 1. Example of median effect transformation. **(a)** Standard Hill plot for three theoretical neutralization curves with high (orange), moderate (black) or low (blue) slope (m) and different IC_{50}s (filled circles). **(b)** Log effect ratio transformation (**Equation (1)**) of the same curves in panel **a**. Slope is indicated by the angle of the line relative to x-axis and IC_{50} is indicated by the x-intercept (filled circles). (**box**) Description of mathematical parameters used for median effect fitting.

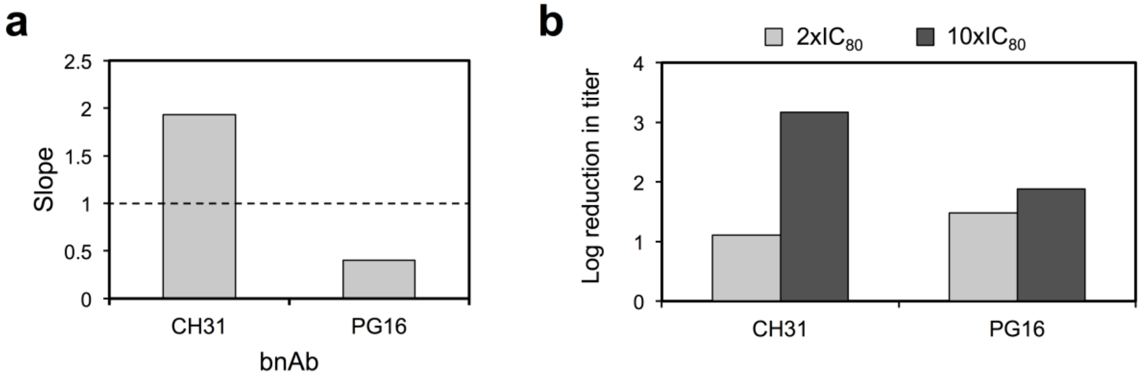

Supplementary Figure 2. Validation of median effect extrapolations to extreme neutralization levels. (**a**) Slopes of CH31 and PG16 against Env Ce1176 from standard neutralization assay. (**b**) Log reductions in viral titer using 2x and 10x the IC_{80} concentrations of CH31 and PG16 determined from titer reduction assay (**Methods**). A greater reduction in titer was observed for PG16 at $2xIC_{80}$, while a more therapeutically relevant reduction in titer was observed for CH31 at $10xIC_{80}$.

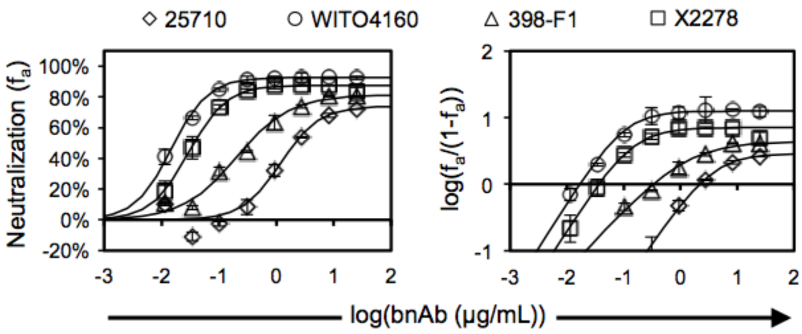

Supplementary Figure 3. Examples of neutralization plateaus. Hill (left) and median effect plots (right) of neutralization for representative examples of Envs where plateaus of neutralization were observed with the V2 glycan bnAb CH01 (**Supplementary Table 2**). Symbols show experimental results and solid lines indicate the median effect predictions after fitting to **Equation (5)** (**Methods**). Bars show mean and standard deviation from two replicates.

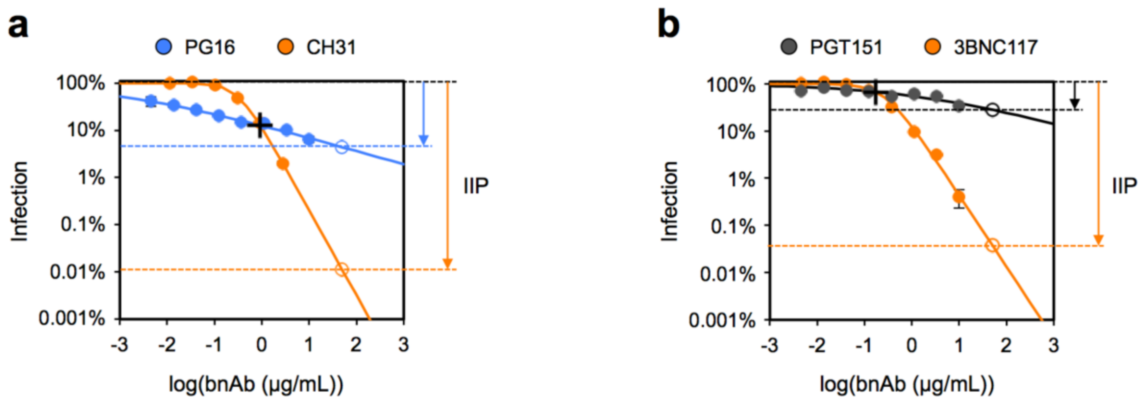

Supplementary Figure 4. Illustrative example of IIP. Residual infection at increasing concentrations of **(a)** PG16 and CH31 and **(b)** PGT151 and 3BNC117 against Env 25710 are shown on a log scale (filled circles). IIP describes the log reduction in infection at a given concentration. Extrapolated residual infection at 50μg/mL is shown as hollow circles and the corresponding log reduction in infection is illustrated by the arrows. Error bars indicate standard deviation of two replicates.

Supplementary Table 1. HIV Envelope Panels.

Global Panel								
**Env**	**Tier**	**Subtype**	**Country**	**Year**	**Fiebig Stage**	**Mode of Transmission**	**GenBank**	**Ref**
TRO11	2	B	Italy	1995	III	M-M	AY835445	1, 2
25710	2	C	India	1999	V	F-M	EF117271	1, 3
398F1	2	A	Tanzania	2001	Not Available	Heterosexual	HM215312	1
CNE8	2	CRF01_AE	China	2006	Chronic Infection	IVDU	HM215427	1, 4
X2278	2	B	Spain	2007	V/VI	Heterosexual	FJ817366	1
BJOX2000	2	CRF07_BC	China	2007	I /II	IVDU	HM215364	1
X1632	2	G	Spain	2004	Chronic Infection	Heterosexual	FJ817370	1, 5
CE1176	2	C	Malawi	2004	I/II	Sexual	FJ444437	1
246F3	2	AC recomb	Tanzania	2001	VI	Heterosexual	HM215279	1
CH119	2	CRF07_BC	China	2004	Chronic Infection	IVDU	EF117261	1
CE0217	2	C	Malawi	2007	V/VI	Sexual	FJ443575	1
CNE55	2	CRF01_AE	China	2007	Chronic Infection	IVDU	HM215418	1, 4
PVO.4	3	B	Italy	1996	III	M-M	AY835444	2
QH0515.1	2	B	Trinidad	1994	V	F-M	AY835440	2
QH0692	2	B	Trinidad	1994	V	F-M	AY835439	2
SC442661	2	B	Trinidad	1995	IV	F-M	AY835441	2
WITO4160	2	B	U.S.	2000	I	F-M	AY835451	2

Supplementary Table 2. Summary of Neutralization Parameters.

Epitope	bnAb	Env	Slope	IC_{50}*	IC_{80}*	IC_{90}*	IC_{99}*	Max†
CD4bs	3BNC117	25710	1.51	0.27	0.68	1.16	5.67	>99%
CD4bs	3BNC117	246-F3	1.51	0.099	0.25	0.42	2.09	>99%
CD4bs	3BNC117	398-F1	1.17	0.074	0.24	0.49	3.78	>99%
CD4bs	3BNC117	BJOX002000	NoN	NoN	NoN	NoN	NoN	NoN
CD4bs	3BNC117	Ce1176	1.24	0.14	0.42	0.8	5.49	>99%
CD4bs	3BNC117	Ce0217	1.44	0.043	0.11	0.2	1.06	>99%
CD4bs	3BNC117	CH119	0.90	5.57	25.92	63.72	910	>99%
CD4bs	3BNC117	CNE55	1.63	0.1	0.23	0.39	1.68	>99%
CD4bs	3BNC117	CNE8	1.26	0.16	0.48	0.92	6.15	>99%
CD4bs	3BNC117	PVO4	N.D.	N.D.	N.D.	N.D.	N.D.	N.D.
CD4bs	3BNC117	QH0515	N.D.	N.D.	N.D.	N.D.	N.D.	N.D.
CD4bs	3BNC117	QH0692	N.D.	N.D.	N.D.	N.D.	N.D.	N.D.
CD4bs	3BNC117	SC422661	N.D.	N.D.	N.D.	N.D.	N.D.	N.D.
CD4bs	3BNC117	TRO11	1.52	0.03	0.074	0.13	0.61	>99%
CD4bs	3BNC117	WITO4160	N.D.	N.D.	N.D.	N.D.	N.D.	N.D.
CD4bs	3BNC117	X1632	0.63	2.5	22.94	83.84	>1000	>99%
CD4bs	3BNC117	X2278	1.32	0.012	0.036	0.066	0.41	>99%
CD4bs	CH31	25710	1.80	0.32	0.69	1.08	4.1	>99%
CD4bs	CH31	246-F3	1.53	0.054	0.13	0.23	1.09	>99%
CD4bs	CH31	398-F1	1.41	0.059	0.16	0.28	1.52	>99%
CD4bs	CH31	BJOX002000	NoN	NoN	NoN	NoN	NoN	NoN
CD4bs	CH31	Ce1176	1.93	1.23	2.52	3.84	13.3	>99%
CD4bs	CH31	Ce0217	1.93	0.059	0.12	0.18	0.64	>99%
CD4bs	CH31	CH119	1.51	1.48	3.72	6.37	31.2	>99%
CD4bs	CH31	CNE55	1.58	0.057	0.14	0.23	1.04	>99%
CD4bs	CH31	CNE8	1.54	0.13	0.31	0.53	2.53	>99%
CD4bs	CH31	PVO4	1.54	0.51	1.24	2.1	9.94	>99%
CD4bs	CH31	QH0515	1.41	0.14	0.37	0.67	3.64	>99%
CD4bs	CH31	QH0692	1.77	0.84	1.84	2.9	11.27	>99%
CD4bs	CH31	SC422661	1.30	0.15	0.44	0.82	5.2	>99%
CD4bs	CH31	TRO11	1.31	0.11	0.32	0.6	3.73	>99%
CD4bs	CH31	WITO4160	1.14	0.13	0.45	0.92	7.53	>99%
CD4bs	CH31	X1632	1.28	0.043	0.13	0.24	1.56	>99%
CD4bs	CH31	X2278	1.54	0.08	0.2	0.33	1.59	>99%
CD4bs	HJ16_22	25710	NoN	NoN	NoN	NoN	NoN	NoN
CD4bs	HJ16_22	246-F3	NoN	NoN	NoN	NoN	NoN	NoN
CD4bs	HJ16_22	398-F1	0.47	21.16	392	>1000	>1000	>99%

Epitope	bnAb	Env	Slope	IC_{50}*	IC_{80}*	IC_{90}*	IC_{99}*	Max†
CD4bs	HJ16_22	BJOX002000	NoN	NoN	NoN	NoN	NoN	NoN
CD4bs	HJ16_22	Ce1176	1.17	0.3	0.97	1.95	15.23	>99%
CD4bs	HJ16_22	Ce0217	1.54	0.053	0.13	0.22	1.05	>99%
CD4bs	HJ16_22	CH119	1.69	0.14	0.31	0.5	2.08	>99%
CD4bs	HJ16_22	CNE55	NoN	NoN	NoN	NoN	NoN	NoN
CD4bs	HJ16_22	CNE8	0.97	11.61	48.74	113	>1000	>99%
CD4bs	HJ16_22	PVO4	N.D.	N.D.	N.D.	N.D.	N.D.	N.D.
CD4bs	HJ16_22	QH0515	N.D.	N.D.	N.D.	N.D.	N.D.	N.D.
CD4bs	HJ16_22	QH0692	N.D.	N.D.	N.D.	N.D.	N.D.	N.D.
CD4bs	HJ16_22	SC422661	N.D.	N.D.	N.D.	N.D.	N.D.	N.D.
CD4bs	HJ16_22	TRO11	1.14	0.078	0.26	0.54	4.42	>99%
CD4bs	HJ16_22	WITO4160	N.D.	N.D.	N.D.	N.D.	N.D.	N.D.
CD4bs	HJ16_22	X1632	NoN	NoN	NoN	NoN	NoN	NoN
CD4bs	HJ16_22	X2278	NoN	NoN	NoN	NoN	NoN	NoN
CD4bs	sCD4	25710	1.32	1.19	3.4	6.31	39.01	>99%
CD4bs	sCD4	246-F3	1.03	12.21	46.7	102	>1000	>99%
CD4bs	sCD4	398-F1	0.87	8.02	39.22	99.29	>1000	>99%
CD4bs	sCD4	BJOX002000	1.04	1.45	5.46	11.87	118	>99%
CD4bs	sCD4	Ce1176	1.06	4.73	17.51	37.66	362	>99%
CD4bs	sCD4	Ce0217	0.72	13.58	93.47	289	>1000	>99%
CD4bs	sCD4	CH119	0.79	15.75	91.54	256	>1000	>99%
CD4bs	sCD4	CNE55	NoN	NoN	NoN	NoN	NoN	NoN
CD4bs	sCD4	CNE8	NoN	NoN	NoN	NoN	NoN	NoN
CD4bs	sCD4	PVO4	N.D.	N.D.	N.D.	N.D.	N.D.	N.D.
CD4bs	sCD4	QH0515	N.D.	N.D.	N.D.	N.D.	N.D.	N.D.
CD4bs	sCD4	QH0692	N.D.	N.D.	N.D.	N.D.	N.D.	N.D.
CD4bs	sCD4	SC422661	N.D.	N.D.	N.D.	N.D.	N.D.	N.D.
CD4bs	sCD4	TRO11	0.46	33.81	685	>1000	>1000	>99%
CD4bs	sCD4	WITO4160	N.D.	N.D.	N.D.	N.D.	N.D.	N.D.
CD4bs	sCD4	X1632	1.24	1.01	3.09	5.93	40.78	>99%
CD4bs	sCD4	X2278	NoN	NoN	NoN	NoN	NoN	NoN
CD4bs	VRC01	25710	1.14	0.5	1.69	3.45	28.52	>99%
CD4bs	VRC01	246-F3	1.56	0.25	0.6	1.02	4.74	>99%
CD4bs	VRC01	398-F1	1.23	0.17	0.51	0.99	6.92	>99%
CD4bs	VRC01	BJOX002000	NoN	NoN	NoN	NoN	NoN	NoN
CD4bs	VRC01	Ce1176	1.50	2.04	5.15	8.85	43.89	>99%
CD4bs	VRC01	Ce0217	1.15	0.21	0.7	1.42	11.36	>99%
CD4bs	VRC01	CH119	1.64	1.19	2.78	4.55	19.71	>99%
CD4bs	VRC01	CNE55	1.44	0.45	1.17	2.05	10.84	>99%
CD4bs	VRC01	CNE8	1.22	0.95	2.96	5.74	40.78	>99%

Epitope	bnAb	Env	Slope	IC_{50} *	IC_{80} *	IC_{90} *	IC_{99} *	Max†
CD4bs	VRC01	PVO4	1.53	0.8	1.98	3.36	16.04	>99%
CD4bs	VRC01	QH0515	1.43	1	2.65	4.68	25.17	>99%
CD4bs	VRC01	QH0692	1.47	1.13	2.9	5.03	25.61	>99%
CD4bs	VRC01	SC422661	1.34	0.11	0.32	0.58	3.48	>99%
CD4bs	VRC01	TRO11	1.48	0.52	1.32	2.3	11.66	>99%
CD4bs	VRC01	WITO4160	1.37	0.23	0.64	1.15	6.66	>99%
CD4bs	VRC01	X1632	0.71	0.15	1.06	3.29	94.16	>99%
CD4bs	VRC01	X2278	1.06	0.066	0.25	0.53	5.14	>99%
gp120/41	PGT151	25710	0.29	1.93	231	>1000	>1000	>99%
gp120/41	PGT151	246-F3	0.38	0.009	0.34	2.88	>1000	76.2%
gp120/41	PGT151	398-F1	0.88	0.005	0.024	0.062	0.95	>99%
gp120/41	PGT151	BJOX002000	1.13	0.019	0.066	0.14	1.14	>99%
gp120/41	PGT151	Ce1176	0.98	0.009	0.037	0.084	0.98	>99%
gp120/41	PGT151	Ce0217	NoN	NoN	NoN	NoN	NoN	NoN
gp120/41	PGT151	CH119	0.58	0.029	0.32	1.29	79.75	93.8%
gp120/41	PGT151	CNE55	NoN	NoN	NoN	NoN	NoN	NoN
gp120/41	PGT151	CNE8	0.72	15.71	109	339	>1000	>99%
gp120/41	PGT151	PVO4	N.D.	N.D.	N.D.	N.D.	N.D.	N.D.
gp120/41	PGT151	QH0515	N.D.	N.D.	N.D.	N.D.	N.D.	N.D.
gp120/41	PGT151	QH0692	N.D.	N.D.	N.D.	N.D.	N.D.	N.D.
gp120/41	PGT151	SC422661	N.D.	N.D.	N.D.	N.D.	N.D.	N.D.
gp120/41	PGT151	TRO11	0.88	12.5	60.23	151	>1000	>99%
gp120/41	PGT151	WITO4160	N.D.	N.D.	N.D.	N.D.	N.D.	N.D.
gp120/41	PGT151	X1632	0.25	0.97	247	>1000	>1000	>99%
gp120/41	PGT151	X2278	0.88	0.024	0.12	0.29	4.5	>99%
HM cluster	2G12	25710	1.03	46.89	179	393	>1000	>99%
HM cluster	2G12	246-F3	NoN	NoN	NoN	NoN	NoN	NoN
HM cluster	2G12	398-F1	NoN	NoN	NoN	NoN	NoN	NoN
HM cluster	2G12	BJOX002000	NoN	NoN	NoN	NoN	NoN	NoN
HM cluster	2G12	Ce1176	NoN	NoN	NoN	NoN	NoN	NoN
HM cluster	2G12	Ce0217	NoN	NoN	NoN	NoN	NoN	NoN
HM cluster	2G12	CH119	NoN	NoN	NoN	NoN	NoN	NoN
HM cluster	2G12	CNE55	NoN	NoN	NoN	NoN	NoN	NoN
HM cluster	2G12	CNE8	NoN	NoN	NoN	NoN	NoN	NoN
HM cluster	2G12	PVO4	0.99	2.65	10.69	24.2	271	>99%
HM cluster	2G12	QH0515	1.47	0.06	0.15	0.27	1.36	>99%
HM cluster	2G12	QH0692	1.36	3.55	9.87	17.93	105	>99%
HM cluster	2G12	SC422661	1.08	3.28	11.89	25.24	234	94.5%
HM cluster	2G12	TRO11	0.98	0.27	1.13	2.57	29.38	>99%
HM cluster	2G12	WITO4160	0.91	2.11	9.72	23.76	334	>99%

Epitope	bnAb	Env	Slope	IC_{50} *	IC_{80} *	IC_{90} *	IC_{99} *	Max†
HM cluster	2G12	X1632	NoN	NoN	NoN	NoN	NoN	NoN
HM cluster	2G12	X2278	1.22	0.24	0.75	1.46	10.43	>99%
MPER	10E8	25710	0.63	0.015	0.13	0.49	22.7	>99%
MPER	10E8	246-F3	0.75	0.38	2.43	7.21	180	>99%
MPER	10E8	398-F1	0.72	0.54	3.71	11.41	316	>99%
MPER	10E8	BJOX002000	0.81	0.45	2.52	6.87	133	>99%
MPER	10E8	Ce1176	1.04	0.32	1.2	2.62	26.36	>99%
MPER	10E8	Ce0217	0.75	0.2	1.27	3.75	91.28	>99%
MPER	10E8	CH119	0.58	0.31	3.33	13.45	835	>99%
MPER	10E8	CNE55	0.65	0.18	1.56	5.49	226	>99%
MPER	10E8	CNE8	0.68	0.021	0.16	0.53	18.01	>99%
MPER	10E8	PVO4	N.D.	N.D.	N.D.	N.D.	N.D.	N.D.
MPER	10E8	QH0515	N.D.	N.D.	N.D.	N.D.	N.D.	N.D.
MPER	10E8	QH0692	N.D.	N.D.	N.D.	N.D.	N.D.	N.D.
MPER	10E8	SC422661	N.D.	N.D.	N.D.	N.D.	N.D.	N.D.
MPER	10E8	TRO11	0.87	0.026	0.13	0.32	5.05	>99%
MPER	10E8	WITO4160	N.D.	N.D.	N.D.	N.D.	N.D.	N.D.
MPER	10E8	X1632	1.01	0.43	1.71	3.81	40.6	>99%
MPER	10E8	X2278	0.83	0.35	1.85	4.95	90.05	>99%
MPER	2F5	25710	NoN	NoN	NoN	NoN	NoN	NoN
MPER	2F5	246-F3	0.88	1.23	5.9	14.77	223	>99%
MPER	2F5	398-F1	0.72	9.64	66.25	205	>1000	>99%
MPER	2F5	BJOX002000	NoN	NoN	NoN	NoN	NoN	NoN
MPER	2F5	Ce1176	NoN	NoN	NoN	NoN	NoN	NoN
MPER	2F5	Ce0217	NoN	NoN	NoN	NoN	NoN	NoN
MPER	2F5	CH119	NoN	NoN	NoN	NoN	NoN	NoN
MPER	2F5	CNE55	0.66	1.34	10.87	37.04	>1000	>99%
MPER	2F5	CNE8	1.06	3.2	11.9	25.65	249	>99%
MPER	2F5	PVO4	N.D.	N.D.	N.D.	N.D.	N.D.	N.D.
MPER	2F5	QH0515	N.D.	N.D.	N.D.	N.D.	N.D.	N.D.
MPER	2F5	QH0692	N.D.	N.D.	N.D.	N.D.	N.D.	N.D.
MPER	2F5	SC422661	N.D.	N.D.	N.D.	N.D.	N.D.	N.D.
MPER	2F5	TRO11	NoN	NoN	NoN	NoN	NoN	NoN
MPER	2F5	WITO4160	N.D.	N.D.	N.D.	N.D.	N.D.	N.D.
MPER	2F5	X1632	0.84	2.45	12.8	33.66	588	>99%
MPER	2F5	X2278	0.87	16.17	78.91	199	>1000	>99%
MPER	4E10	25710	1.10	1.15	4.03	8.42	74.26	>99%
MPER	4E10	246-F3	0.72	3.74	25.43	78.04	>1000	>99%
MPER	4E10	398-F1	0.76	12.88	78.87	228	>1000	>99%
MPER	4E10	BJOX002000	0.54	4.95	63.61	283	>1000	>99%

Epitope	bnAb	Env	Slope	IC_{50} *	IC_{80} *	IC_{90} *	IC_{99} *	Max†
MPER	4E10	Ce1176	1.00	6.72	26.88	60.51	666	>99%
MPER	4E10	Ce0217	0.81	1.37	7.53	20.42	389	>99%
MPER	4E10	CH119	1.26	6.45	19.38	36.88	247	>99%
MPER	4E10	CNE55	0.60	2.98	30.54	119	>1000	>99%
MPER	4E10	CNE8	0.97	7	29.42	68.17	818	>99%
MPER	4E10	PVO4	N.D.	N.D.	N.D.	N.D.	N.D.	N.D.
MPER	4E10	QH0515	N.D.	N.D.	N.D.	N.D.	N.D.	N.D.
MPER	4E10	QH0692	N.D.	N.D.	N.D.	N.D.	N.D.	N.D.
MPER	4E10	SC422661	N.D.	N.D.	N.D.	N.D.	N.D.	N.D.
MPER	4E10	TRO11	0.88	1.19	5.73	14.33	216	>99%
MPER	4E10	WITO4160	N.D.	N.D.	N.D.	N.D.	N.D.	N.D.
MPER	4E10	X1632	0.64	2.62	22.86	81.19	>1000	>99%
MPER	4E10	X2278	0.57	11.31	127	524	>1000	>99%
V2-glycan	CH01	25710	1.28	1.21	3.55	6.68	43.35	74.1%
V2-glycan	CH01	246-F3	0.82	0.74	4.02	10.85	204	82.9%
V2-glycan	CH01	398-F1	0.93	0.2	0.89	2.14	28.55	81.2%
V2-glycan	CH01	BJOX002000	0.41	29.06	881	>1000	>1000	>99%
V2-glycan	CH01	Ce1176	NoN	NoN	NoN	NoN	NoN	NoN
V2-glycan	CH01	Ce0217	0.87	0.28	1.38	3.51	55.22	93.8%
V2-glycan	CH01	CH119	1.22	1.38	4.31	8.36	59.54	92.3%
V2-glycan	CH01	CNE55	NoN	NoN	NoN	NoN	NoN	NoN
V2-glycan	CH01	CNE8	NoN	NoN	NoN	NoN	NoN	NoN
V2-glycan	CH01	PVO4	N.D.	N.D.	N.D.	N.D.	N.D.	N.D.
V2-glycan	CH01	QH0515	N.D.	N.D.	N.D.	N.D.	N.D.	N.D.
V2-glycan	CH01	QH0692	N.D.	N.D.	N.D.	N.D.	N.D.	N.D.
V2-glycan	CH01	SC422661	N.D.	N.D.	N.D.	N.D.	N.D.	N.D.
V2-glycan	CH01	TRO11	NoN	NoN	NoN	NoN	NoN	NoN
V2-glycan	CH01	WITO4160	N.D.	N.D.	N.D.	N.D.	N.D.	N.D.
V2-glycan	CH01	X1632	0.63	0.58	5.21	18.8	838	73.2%
V2-glycan	CH01	X2278	1.34	0.03	0.084	0.15	0.92	87.6%
V2-glycan	PG16	25710	0.29	0.001	0.14	2.37	>1000	>99%
V2-glycan	PG16	246-F3	NoN	NoN	NoN	NoN	NoN	NoN
V2-glycan	PG16	398-F1	NoN	NoN	NoN	NoN	NoN	NoN
V2-glycan	PG16	BJOX002000	NoN	NoN	NoN	NoN	NoN	NoN
V2-glycan	PG16	Ce1176	0.40	0.001	0.024	0.18	70.36	>99%
V2-glycan	PG16	Ce0217	1.69	0.002	0.004	0.006	0.024	>99%
V2-glycan	PG16	CH119	0.61	0.53	5.19	19.8	>1000	>99%
V2-glycan	PG16	CNE55	0.33	1.29	91.36	>1000	>1000	>99%
V2-glycan	PG16	CNE8	0.37	0.5	21.17	188	>1000	>99%
V2-glycan	PG16	PVO4	N.D.	N.D.	N.D.	N.D.	N.D.	N.D.

Epitope	bnAb	Env	Slope	IC_{50} *	IC_{80} *	IC_{90} *	IC_{99} *	Max†
V2-glycan	PG16	QH0515	N.D.	N.D.	N.D.	N.D.	N.D.	N.D.
V2-glycan	PG16	QH0692	N.D.	N.D.	N.D.	N.D.	N.D.	N.D.
V2-glycan	PG16	SC422661	N.D.	N.D.	N.D.	N.D.	N.D.	N.D.
V2-glycan	PG16	TRO11	NoN	NoN	NoN	NoN	NoN	NoN
V2-glycan	PG16	WITO4160	N.D.	N.D.	N.D.	N.D.	N.D.	N.D.
V2-glycan	PG16	X1632	0.34	0.014	0.82	8.8	>1000	>99%
V2-glycan	PG16	X2278	0.79	0.002	0.012	0.033	0.7	>99%
V2-glycan	PG9	25710	0.96	0.04	0.17	0.4	4.87	98.3%
V2-glycan	PG9	246-F3	1.12	0.022	0.076	0.16	1.33	>99%
V2-glycan	PG9	398-F1	NoN	NoN	NoN	NoN	NoN	NoN
V2-glycan	PG9	BJOX002000	0.68	0.079	0.6	1.99	67.89	>99%
V2-glycan	PG9	Ce1176	0.86	0.006	0.028	0.072	1.18	>99%
V2-glycan	PG9	Ce0217	1.13	0.005	0.018	0.036	0.3	>99%
V2-glycan	PG9	CH119	1.33	0.51	1.45	2.67	16.27	>99%
V2-glycan	PG9	CNE55	0.56	0.077	0.93	4.01	301	>99%
V2-glycan	PG9	CNE8	0.69	0.46	3.45	11.25	371	>99%
V2-glycan	PG9	PVO4	N.D.	N.D.	N.D.	N.D.	N.D.	N.D.
V2-glycan	PG9	QH0515	N.D.	N.D.	N.D.	N.D.	N.D.	N.D.
V2-glycan	PG9	QH0692	N.D.	N.D.	N.D.	N.D.	N.D.	N.D.
V2-glycan	PG9	SC422661	N.D.	N.D.	N.D.	N.D.	N.D.	N.D.
V2-glycan	PG9	TRO11	0.90	17.7	82.72	204	>1000	>99%
V2-glycan	PG9	WITO4160	N.D.	N.D.	N.D.	N.D.	N.D.	N.D.
V2-glycan	PG9	X1632	0.77	0.11	0.64	1.85	42.16	89.2%
V2-glycan	PG9	X2278	0.94	0.012	0.054	0.13	1.64	>99%
V3-glycan	10-1074	25710	1.40	0.08	0.21	0.38	2.12	>99%
V3-glycan	10-1074	246-F3	NoN	NoN	NoN	NoN	NoN	NoN
V3-glycan	10-1074	398-F1	1.13	0.011	0.036	0.073	0.61	>99%
V3-glycan	10-1074	BJOX002000	1.46	0.017	0.044	0.076	0.4	>99%
V3-glycan	10-1074	Ce1176	1.76	0.029	0.063	0.099	0.39	>99%
V3-glycan	10-1074	Ce0217	1.11	0.008	0.029	0.061	0.52	>99%
V3-glycan	10-1074	CH119	1.51	0.026	0.064	0.11	0.54	>99%
V3-glycan	10-1074	CNE55	NoN	NoN	NoN	NoN	NoN	NoN
V3-glycan	10-1074	CNE8	NoN	NoN	NoN	NoN	NoN	NoN
V3-glycan	10-1074	PVO4	N.D.	N.D.	N.D.	N.D.	N.D.	N.D.
V3-glycan	10-1074	QH0515	N.D.	N.D.	N.D.	N.D.	N.D.	N.D.
V3-glycan	10-1074	QH0692	N.D.	N.D.	N.D.	N.D.	N.D.	N.D.
V3-glycan	10-1074	SC422661	N.D.	N.D.	N.D.	N.D.	N.D.	N.D.
V3-glycan	10-1074	TRO11	1.23	0.019	0.06	0.12	0.82	>99%
V3-glycan	10-1074	WITO4160	N.D.	N.D.	N.D.	N.D.	N.D.	N.D.
V3-glycan	10-1074	X1632	NoN	NoN	NoN	NoN	NoN	NoN

Epitope	bnAb	Env	Slope	IC_{50} *	IC_{80} *	IC_{90} *	IC_{99} *	Max†
V3-glycan	10-1074	X2278	1.74	0.031	0.07	0.11	0.44	>99%
V3-glycan	PGT121	25710	1.32	0.028	0.081	0.15	0.93	>99%
V3-glycan	PGT121	246-F3	NoN	NoN	NoN	NoN	NoN	NoN
V3-glycan	PGT121	398-F1	1.74	0.03	0.067	0.11	0.42	>99%
V3-glycan	PGT121	BJOX002000	1.40	0.024	0.064	0.11	0.64	>99%
V3-glycan	PGT121	Ce1176	1.58	0.019	0.044	0.074	0.34	>99%
V3-glycan	PGT121	Ce0217	1.17	0.004	0.012	0.024	0.18	>99%
V3-glycan	PGT121	CH119	0.92	0.016	0.072	0.17	2.38	>99%
V3-glycan	PGT121	CNE55	NoN	NoN	NoN	NoN	NoN	NoN
V3-glycan	PGT121	CNE8	NoN	NoN	NoN	NoN	NoN	NoN
V3-glycan	PGT121	PVO4	N.D.	N.D.	N.D.	N.D.	N.D.	N.D.
V3-glycan	PGT121	QH0515	N.D.	N.D.	N.D.	N.D.	N.D.	N.D.
V3-glycan	PGT121	QH0692	N.D.	N.D.	N.D.	N.D.	N.D.	N.D.
V3-glycan	PGT121	SC422661	N.D.	N.D.	N.D.	N.D.	N.D.	N.D.
V3-glycan	PGT121	TRO11	1.44	0.014	0.036	0.062	0.33	>99%
V3-glycan	PGT121	WITO4160	N.D.	N.D.	N.D.	N.D.	N.D.	N.D.
V3-glycan	PGT121	X1632	NoN	NoN	NoN	NoN	NoN	NoN
V3-glycan	PGT121	X2278	1.33	0.023	0.065	0.12	0.73	>99%
V3-glycan	PGT128	25710	1.57	0.029	0.07	0.12	0.54	>99%
V3-glycan	PGT128	246-F3	1.52	0.007	0.017	0.029	0.14	>99%
V3-glycan	PGT128	398-F1	1.33	0.005	0.014	0.026	0.16	>99%
V3-glycan	PGT128	BJOX002000	1.85	0.062	0.13	0.2	0.74	97.4%
V3-glycan	PGT128	Ce1176	NoN	NoN	NoN	NoN	NoN	NoN
V3-glycan	PGT128	Ce0217	0.94	0.063	0.28	0.66	8.63	>99%
V3-glycan	PGT128	CH119	1.80	0.048	0.1	0.16	0.62	>99%
V3-glycan	PGT128	CNE55	NoN	NoN	NoN	NoN	NoN	NoN
V3-glycan	PGT128	CNE8	1.44	0.03	0.079	0.14	0.73	>99%
V3-glycan	PGT128	PVO4	N.D.	N.D.	N.D.	N.D.	N.D.	N.D.
V3-glycan	PGT128	QH0515	N.D.	N.D.	N.D.	N.D.	N.D.	N.D.
V3-glycan	PGT128	QH0692	N.D.	N.D.	N.D.	N.D.	N.D.	N.D.
V3-glycan	PGT128	SC422661	N.D.	N.D.	N.D.	N.D.	N.D.	N.D.
V3-glycan	PGT128	TRO11	1.51	0.028	0.069	0.12	0.58	>99%
V3-glycan	PGT128	WITO4160	N.D.	N.D.	N.D.	N.D.	N.D.	N.D.
V3-glycan	PGT128	X1632	NoN	NoN	NoN	NoN	NoN	NoN
V3-glycan	PGT128	X2278	1.35	0.012	0.033	0.061	0.36	>99%

N.D., Not Done.

NoN, Non Neutralized. Neutralization ≥50% was not observed within the range of bnAb concentrations assayed or median effect predicted IC_{50} was ≥50μg/mL.

* Potencies are determined from median effect fit parameters in units of μg/mL

† Fitted maximum neutralization (see **Methods**)

SUPPLEMENTARY REFERENCES

1. deCamp, A. *et al.* Global panel of HIV-1 Env reference strains for standardized assessments of vaccine-elicited neutralizing antibodies. *J Virol* **88**, 2489-2507, doi:10.1128/JVI.02853-13 (2014).
2. Li, M. *et al.* Human immunodeficiency virus type 1 env clones from acute and early subtype B infections for standardized assessments of vaccine-elicited neutralizing antibodies. *J Virol* **79**, 10108-10125, doi:10.1128/JVI.79.16.10108-10125.2005 (2005).
3. Kulkarni, SS. *et al*. Highly complex neutralization determinants on a monophyletic lineage of newly transmitted subtype C HIV-1 Env clones from India. *Virology* **385**, 505-520, doi:10.1016/j.virol.2008.12.032 (2009).
4. Shang, H. *et al.* Genetic and neutralization sensitivity of diverse HIV-1 env clones from chronically infected patients in China. *J. Biol. Chem.* **286**, 14531-14541, doi:10.1074/jbc.M111.224527 (2011).
5. Revilla, A. *et al.* Construction and phenotypic characterization of HIV type 1 functional envelope clones of subtypes G and F. *AIDS Res Hum Retroviruses* **27**, 889-901, doi:10.1089/AID.2010.0177 (2011).
